# Seeking Systematicity in Variation: Theoretical and Methodological Considerations on the “Variety” Concept

**DOI:** 10.3389/fpsyg.2018.00385

**Published:** 2018-03-26

**Authors:** Anne-Sophie Ghyselen, Gunther De Vogelaer

**Affiliations:** ^1^Research Centre for Multilingual Practices and Language Learning in Society, Department of Linguistics, Ghent University, Ghent, Belgium; ^2^Institut für Niederländische Philologie, Westfälische Wilhelms-Universität Münster, Münster, Germany

**Keywords:** variety, structured heterogeneity, style-shifting, polylanguaging, prototype theory, competing grammars, Dutch

## Abstract

One centennial discussion in linguistics concerns whether languages, or linguistic systems, are, essentially, homogeneous or rather show “structured heterogeneity.” In this contribution, the question is addressed whether and how sociolinguistically defined systems (or ‘varieties’) are to be distinguished in a heterogeneous linguistic landscape: to what extent can structure be found in the myriads of language variants heard in everyday language use? We first elaborate on the theoretical importance of this ‘variety question’ by relating it to current approaches from, among others, generative linguistics (competing grammars), sociolinguistics (style-shifting, polylanguaging), and cognitive linguistics (prototype theory). Possible criteria for defining and detecting varieties are introduced, which are subsequently tested empirically, using a self-compiled corpus of spoken Dutch in West Flanders (Belgium). This empirical study demonstrates that the speech repertoire of the studied West Flemish speakers consists of four varieties, viz. a fairly stable dialect variety, a more or less virtual standard Dutch variety, and two intermediate varieties, which we will label ‘cleaned-up dialect’ and ‘substandard.’ On the methodological level, this case-study underscores the importance of speech corpora comprising both inter- and intra-speaker variation on the one hand, and the merits of triangulating qualitative and quantitative approaches on the other.

## Introduction

Contemporary linguistic analysis almost inevitably builds on implicit or explicit assumptions about the structure of linguistic variation. Whereas some approaches for instance assume that speakers select language variants from different systems or ‘grammars’ of language features (cf. concepts such as code switching or multilingualism), others assume that speakers have one ‘variation space’ at their disposal, comprising a whole array of features, that are selected strategically (cf. concepts such as style-shifting or translanguaging). A theoretical tension between these positions can be traced back at least to the 1960s, when both Bright (1966) and Labov (1969b) criticized the then canonical classification of inter- and intra-speaker variation as ‘free’ and demanded more scholarly attention for the “orderly heterogeneity” (Weinreich et al., 1968, p. 100) within language varieties. The selection of variants was shown to be constrained by both language internal factors (such as phonetic environment) and language external ones (such as speakers' age, gender, and regional and social identity). While this idea is by now generally acknowledged in both generative and usage-based approaches to language, the cognitive mechanisms underpinning selection processes remain unclear. For instance, there is still a debate about the ontological status of the linguistic system (Geeraerts, 2010), with differing opinions on the degree to which systems or varieties can actually be distinguished in the heterogeneity of everyday language. The current paper addresses this topic (henceforth called the variety problem) by critically reviewing the variety concept within linguistics and the way in which it is methodologically implemented. We first elaborate on the theoretical importance of the variety problem by tracing back its roots in linguistic history and relating it to current approaches from, among others, generative linguistics, cognitive linguistics, and sociolinguistics. Possible criteria for defining and detecting varieties are subsequently introduced, which operate on the level of both the individual language user and the speech community. Finally, we test these criteria empirically, using a self-compiled corpus of spoken Dutch in West Flanders (Belgium).

## The variety problem

### Relevance in past and present linguistics

The question how much systematicity can be found in everyday linguistic heterogeneity can be considered essential to the development of sociolinguistics in the 1960s and 1970s. At that time, generative linguists were primarily concerned with “an ideal speaker-listener, in a completely homogeneous speech-community” (Chomsky, 1965, p. 3), considering intraspeaker variation the result of optional rules. Pioneers in sociolinguistics, however, dismissed the idea of free variation within the linguistic system, emphasizing the structures underlying variation. Building on the notion of optionality, Labov (1969a) introduced the concept of the variable rule, which allowed probabilistic modeling of the discrete choices speakers make, using language internal and language external factors as predictors. On a methodological level, Labov (1969a, p. 759) especially insisted on moving away from introspective studies focusing on individuals, toward quantitative investigations of larger samples of the speech community. The grammar of the speech community would after all be “more regular and systematic than the behavior of any one individual” (Labov, 1972a, p. 124). In its focus on structured heterogeneity and community grammars, Labovian sociolinguistics stands diametrically opposed to the idealized object of (early) Chomskyan linguistics, being allegedly homogeneous grammars as used by individual speaker-listeners.

The development of the sociolinguistic paradigm brought the variety question into prominence, but the issue has clear precedents earlier in the history of linguistics. Dialect geography, for instance, has intensely debated whether dialect areas representing separate systems can be distinguished. Already in 1886, Wenker observed that isoglosses are too disparate to allow dividing the variationist landscape into dialect regions, a view also shared by Paris (1888):

*There are in reality no dialects; there are only linguistic features which are combined in multiple ways, to such a degree that the speech of one location will contain a number of features which also occur, for example, in the speech of everyone in the four nearest adjacent places, and a number of features which will differ in the speech of each of them (Paris, 1888, p. 163, own translation ASG & GDV)*.

The remarks by Wenker (1886) and Paris (1888) indicate that nineteenth century dialectologists were well aware of the difficulty of delineating dialect systems, even if these systems are conceived as geographical entities rather than cognitive ones. As Auer (2004, p. 152) remarks, however, popular representations of dialectal variation have always sliced the dialectal landscape into demarcated dialect regions. In attempts to make such maps more accurate, transition areas are commonly distinguished (cf. Wiesinger, 1983; Taeldeman, 2009, p. 359), and borders between areas are deconstructed as bundles of isoglosses. With the development of social dialectology, even the very concept of isoglosses has been problematized (cf. Chambers and Trudgill, 1998, p. 104–118), as seemingly abrupt borders between dialects with variant A and B in reality appear to show a more gradual transition, involving a transitional area in which both A and B are found. Consequently, current dialect geography has developed more advanced tools than isoglosses to model such variation, usually involving probabilistic modeling (Heeringa and Nerbonne, 2001; Pickl, 2013).

The variety question is not only relevant to dialectology, but also constitutes an important topic in many other linguistic disciplines, where the tension between the homogeneity ideal and conceptions assuming structured heterogeneity is equally palpable. In analyses of intraspeaker variation within the Universal Grammar framework, for instance, the idea of homogenous systems underlying variation lives on most clearly in the competing grammars approach (e.g., Kroch, 1989; Lightfoot, 1999). Adopting this idea, Yang (2000) suggests that an individual's variable linguistic behavior can be modeled as a statistical distribution of multiple idealized grammars. A weaker version of this perspective can be found in the sociolinguistic concept of code-switching, which implies that speakers switch between codes or systems depending on situation or speaker intention (cf. Gumperz, 1977)^1^. Opposed to the concepts of competing grammars and code-switching are the notions of *style-shifting* and *translanguaging*. Compared to code-switching, style-shifting implies more “slippery” boundaries between types of language use and weaker co-occurrence constraints between features (Giacalone Ramat, 1995, p. 46; Ervin-Tripp, 2002, p. 49). A more radical version of the style-shifting concept can be found in the increasingly popular notion of *translanguaging*, which maximally dispenses with the idea of speakers using different linguistic systems, even when these are not genealogically related. Advocates of translanguaging consider “the language practices of bilinguals not as two autonomous language systems as has been traditionally the case, but as one linguistic repertoire with features that have been societally constructed as belonging to two separate languages” (García and Wei, 2014, p. 2).

Concepts like dialect continua, dialect-to-standard continua (or diaglossia, cf. Auer, 2005) and translanguaging have gained prominence in linguistics and challenge notions like dialect, language variety and even language. Lenz (2010, p. 302), however, pertinently remarks that “the continuum posited by linguists stands in stark contrast to speakers' very clear ideas about a structured variety spectrum,” leading to the legitimate question whether (and how) speakers in for instance diaglossic communities cognitively distinguish between multiple underlying systems, even when they commonly mix elements from them. Similarly, in translanguaging approaches, the problem remains that language users, who are assumed to combine mere individual variants, often have clear ideas about differences between languages (or language varieties; cf. Geeraerts, 2010, p. 238 on structure as a cognitive fact). These ideas are undeniably socially and culturally determined—and as such more typical of, in Le Page and Tabouret-Keller's (1985) terms, focused communities rather than diffuse ones (see also Trudgill, 1986, p. 85–86 for discussion)—but they do influence ideologies and language practices (cf. Makoni and Pennycook, 2007, p. 21 on the effects of, in their terms, “language inventions”).

### The variety question and the changing sociolinguistic landscape

This article investigates the implications of the variety question for research on changing sociolinguistic landscapes in contemporary Europe, where phenomena are observed like dialect shift and dialect leveling (see e.g., Hinskens et al., 2005, p. 11; Vandekerckhove, 2009), as well as more recent developments relating to the position of the standard language. Dialect leveling refers to the structural processes whereby variation both within and between dialects is lost, whereas dialect shift encompasses the gradual loss of a dialect's communicative functions, i.e., its abandonment in favor of another language variety. Similarly, studies on standard language dynamics distinguish between structural and functional processes. On the structural level, the term demotization—coined by Mattheier (1997)—refers to the process of standard language change whereby ‘the “standard ideology” as such remains intact, while the valorization of ways of speaking changes’ (Coupland and Kristiansen, 2011, p. 28). This process hence assumes change within the existing standard variety. A case in point are the Netherlands, where lowered realizations of the standard Dutch diphthongs [ε.i], [œ.y], and [ɔ.u] are gaining prestige (Van Bezooijen, 2001). Many of the alleged examples of demotization can be analyzed alternatively, however, as the result of the emergence of new varieties and subsequent functional change, i.e., the loss of communicative functions on the part of the former standard. In the case of destandardization, ‘the established standard language loses its position as the one and only “best language”’ (Coupland and Kristiansen, 2011, p. 28), and is replaced with other varieties in some communicative domains. Such a scenario of ‘standard language shift’ typically implies that the Standard Language Ideology—the idea that one type of language is inherently better and prestigious than others—loses ground.

The distinguished processes (dialect shift vs dialect leveling, destandardization vs. demotization) all subscribe to the idea that multiple systems, such as dialect, intermediate language, and standard language, co-exist, but vary in terms of whether they capture the changes in point as system-internal or as a result of a dynamic interplay between varieties. Crucially, the question arises how one can discriminate between processes of linguistic change within an existing variety and the emergence or loss of a variety (Lenz, 2010, p. 295). This question is especially relevant in diaglossic language communities (such as Dutch, but also German, Danish, Italian, … language areas), where transitions between varieties are smooth and in which the traditional dialects tend to be replaced with “intermediate” language use. This intermediate language use can then either be seen as the online result of speakers combining dialectal and standard variants (which assumes the knowledge of two separate linguistic systems on the speakers' part), or as a newly emerged variety in its own right, which replaces the traditional dialect (an instance of dialect shift). However, as this intermediate language use is marked by a combination of dialect and standard features, one might as well hypothesize that the dialect has leveled. Here again, the variety question proves relevant.

### Relativizing systematicity

In addressing the variety question, concepts like translanguaging as opposed to multilingualism present rather extreme positions. As an example of an intermediate position, Auer's typology of dialect/standard constellations relates the answer to the variety question to the language context under study. Whereas *diaglossic* dialect/standard constellations, characterized by a continuum of intermediate variants between base dialect and standard language and style-shifting behavior, would usually be marked by “non-discrete structures” (Auer, 2005, p. 22–23), speakers in *diglossic* communities would clearly distinguish between dialect and standard and code-switch between these. In his view, whether or not variety boundaries exist, varies from context to context and can only be established after careful empirical study (Auer, 2011, p. 491).

A middle ground can also be found in approaches linking the variety question to prototype theory (Jørgensen, 2008; Kristiansen, 2008; Geeraerts, 2010; Pickl, 2013; Geeraerts and Kristiansen, 2015). In such approaches, linguistic categories (a sound, a word, a grammatical rule,…) typically show graded membership, with central and peripheral members. Variety categories too, may display smooth and gradual transitions into one another (Kristiansen, 2008). As such, linguists' problems in delineating varieties compare very well to laymen's handling of graded categories, like colors:

*Some people deny that RP exists. This seems to me like denying that the colour red exists. We may have difficulty in circumscribing it, in deciding whether particular shades verging on pink or orange count as ‘red’, ‘near-red’, or ‘non-red’ […]. Similarly we may hesitate about a particular person's speech which might or might not be ‘RP’ or ‘Near-RP’; we may prefer to call it ‘BBC English’, ‘southern British Standard’, ‘General British’, ‘a la-di-la accent’ or even ‘Standard English’, and define it more narrowly or more widely than I have done, but anyone who has grown up in England knows it when he hears a typical instance of it (Wells, 1982, p. 301)*.

Prototype theory offers a way out of such problems, by conceiving of variety categories as prototypes. An account of varieties as prototypes explains why language users tend to perceive different varieties in a more or less uniform way, but, depending on the circumstances, boundaries between categories may also be relatively fluid, and certain instances may be ambiguous as to the category under which they can be subsumed. While prototype theory essentially describes a cognitive process, viz. categorization, it also leaves room for interindividual variation, yielding a socio-cognitive approach that is geared at mapping both social and historical variation, which is seen as the result of accumulating interindividual differences. Still, the question rises how change within a variety can be distinguished from emergence/loss of a variety (cf. supra).

## Criteria for variety status

The question whether or not varieties can be distinguished in language repertoires, is inextricably connected to the interpretation of the term ‘variety.’ The term is firmly rooted as a theoretical concept in variationist linguistics, but its interpretation varies. In what follows, we take stock of criteria used to define and detect varieties.

### Homogeneity, covariance, and stylistic functions

Chomky's homogeneity axiom relates to de Saussure's (1916) conception of a *langue*, which can be described as an independent, homogenous system. This conception in terms of homogeneous varieties is strongly reflected in present-day linguistic practice—see for instance descriptions of the ‘dialect of location x’ or prescriptive standard language dictionaries and grammars. Usage-based approaches to language, however, have questioned the autonomy of linguistic categories commonly assumed in structuralism and Universal Grammar. Rather, they conceive of linguistic categories as emerging from the memorization and the reorganization of concrete linguistic material, and maintain that categories do not exist independently of the stored instances from which they emerge. When applied to language variation, such usage-based approaches highlight the question how varieties or languages emerge from the heterogeneity of speech signals, and the idea of homogeneous linguistic systems becomes illusory (cf. Geeraerts, 2010). Descriptions of homogeneous varieties thus run the risk of being “mere analyst's play” (Willemyns, 1985), and are considered, from a sociolinguistic point of view, theoretical or socio-political constructs (Makoni and Pennycook, 2007; García and Wei, 2014). Schmidt (2005, p. 62–63) goes even further by arguing that the Saussurian conceptualization of everyday linguistic heterogeneity as a complex of homogenous varieties is not only theoretically obsolete, it would also occasion methodological inadequacies, with as prime example the practice of surveying single informants to map “the base dialect” of whole speech communities.

In attempts to align the variety concept with the inherently heterogeneous and dynamic nature of language, without sacrificing the concept of structured linguistic systems, it has been suggested to use covariance as basic criterion to distinguish varieties. Authors such as Weinreich et al. (1968, p. 169) and Berruto (2010) conceptualize varieties as sets of variants strongly correlating in their socio-situative behavior. Important in this perspective is that correlation or covariance does not necessarily imply strict *co-occurrence* (Weinreich et al., 1968, p. 169); one variant can occur in multiple varieties (Berruto, 2010, p. 236). Covariance can be studied at the level of the individual, but most usually, a community perspective is assumed (see e.g., Geeraerts, 2010). Ideally, sets of covarying language features are used in comparable situations by multiple speakers, with similar stylistic functions that can be described on the level of the speech community. Covariance approaches thus abstract away from the variation in everyday language usage and project descriptions of largely homogeneous varieties on it. A crucial difference with the Saussurian variety concept is that cognitively inspired ‘prototype structures’ are assumed behind variation (cf. supra), which, referring to graphic or statistical representations of such structures, are also labeled “clustering tendencies” (Downes, 1984, p. 28) or “concentration areas” (Berruto, 1989).

Methodologically, the covariance criterion implies empirical study of the systematic co-occurrence of groups of linguistic features in the context of external variables (cf. Geeraerts and Kristiansen, 2015, p. 380), such as speech setting, regional background of both speaker and hearer, and the type of interpersonal relation between interlocutors (cf. Bell, 2001). In this context, multivariate statistical techniques, which allow the simultaneous analysis of multiple dependent variables, are indispensable. In the last decades, different multivariate approaches have been applied in linguistic studies on variation structure, such as factor analysis (Nerbonne, 2006; Pickl, 2013), multidimensional scaling (Ruette and Speelman, 2012, 2013), correspondence analysis (Plevoets, 2008; Geeraerts, 2010), and cluster analysis (Lenz, 2006; Nerbonne et al., 2008), all illustrating the added value of studying the interrelation between multiple dependent and independent variables. A big advantage of these techniques is that they are in essence descriptive, and as such allow discovering structures bottom-up. In contrast to hypothesis testing techniques such as logistic regression, the researcher does not need pre-existing hypotheses on categories that might be relevant.

When clusters of language features displaying similar behavior have been detected, multivariate statistical techniques are also convenient to map the stylistic functions of these clusters. As they not only reveal the covariance between language features, but also the correlations with external parameters (for instance situations or speaker type), insight can be gained in the conditions in which (clusters of) features are generally used. Despite the increasing power of statistical analyses, detecting the stylistic functions of variant clusters is still to a considerable extent done on the basis of qualitative data in present-day sociolinguistics. The case-study presented below will for instance illustrate that the way in which language users describe and account for their own language use in specific speech settings often conveys much information on why speakers realize a particular type of language use in a specific setting.

### Cognitive boundaries and emic category status

Schmidt (2005) and Lenz (2010) propose an approach in which covariance is central, but also argue that the perceptions of speakers have to be taken into account when describing varieties (cf. also Auer and Hinskens, 1996; Agha, 2004). In their view, a variety can be identified when (1) a bundle of language variants marked by covariance can be distinguished and when (2) language users experience this type of language use as a variety. A variety in other words needs to have *emic category status* (Auer, 1986), meaning that the social group realizing a certain type of language use should also perceive that type of language use as a separate category. In such a perceptual approach, if a bundle of language features is marked by linguistic cohesion, but not by perceptual or cognitive boundaries, it is not categorized as a variety, but rather as a ‘speech level’ (*Sprechlage*), i.e., a sublevel within a variety.

Factoring in the ontological status of variant clusters has the attractive advantage that it can offer a solution to the demarcation problem encountered in language change studies (cf. supra). Concerning dialect shift, speakers' language use could be argued to be dialect, if they have the intention of speaking dialect and their language is perceived to be dialect within the speech community, irrespective of whether leveling is involved (cf. Ghyselen and Van Keymeulen, 2014). In this perspective, dialect shift occurs when fewer people have the intention of speaking dialect and stop identifying their language use with the local dialect; dialect leveling is at stake when changes are detected in language use widely perceived to be dialect. Similarly, destandardization takes place when speakers no longer intend to speak standard and their language is also not perceived to be standard. In a scenario of demotization, however, changes can be observed in production patterns, but the language use in question is still perceived/intended to be standard.

In contrast to the covariance criterion, which is typically (but not by definition) implemented by using corpora pooling data from several speakers, the criterion of emic category status presupposes a cognitive perspective on language as it relates to internalized knowledge and gives a central role to the individual language user (cf. Johnstone, 2000). Still, since ‘social meaning’ is to a large extent determined, or rather negotiated, on the community level, it seems reasonable to only ascribe variety status when there is enough homogeneity in the perceptions of the speech community; the “sociological fractionation” (Agha, 2004, p. 27) cannot be exuberant. As was the case with the covariance concept, homogeneity still plays a role, even when it is not used as criterion par excellence.

Important to highlight is that emic category status is not easily detectable in empirical research of language use. After all, usage data do not provide any direct access into mental categories and the boundaries between them. According to Lenz (2004), cognitive boundaries become evident in hyperforms, avoidance strategies and sanctions. These phenomena have in common that they are rare^2^. In addition, detecting them often is methodologically challenging, and may require projecting cognitive boundaries on observable linguistic patterns, which is to be avoided when a test is designed exactly to detect those (cf. Ghyselen, 2015, p. 48–49). An alternative way to gain insight in cognitive boundaries is by studying naming practices. Cornips et al. (2015, p. 47) convincingly argue that “language names play a crucial role as a target in ‘enregisterment’ practices where one language (variety) is distinguished from another through speech typification practices.” Hence, names attributed to different types of language use by linguistic laymen can be a valuable means of gaining insight into grassroots categorizations. Labeling practices can be studied by analyzing public discourse (cf. Cornips et al., 2015; Jaspers and Mercelis, 2015), or by means of sociolinguistic interviews (cf. Léglise and Migge, 2006; Lybaert, 2014a; Jaspers and Mercelis, 2015).

### Idiovarietary features

As was discussed above, the covariance perspective allows variants to occur in multiple varieties; covariance does not equal strict co-occurrence. Varieties might, however, be marked by idiovarietary features, i.e., language variants only appearing in one particular variety. These are usually not considered essential for variety status (cf. Berruto, 2010, p. 236), but they do make a variety more recognizable and can hence further emic category status. Building on Labov's distinction between indicators, markers, and stereotypes (cf. Labov, 1972b), one could argue that idiovarietary features are more likely to be markers or stereotypes, as clear contrasts with equivalent variants stimulate a linguistic variant's salience or cognitive prominence (Hickey, 2000). Looking for possible idiovarietary features can be done both by means of corpora and, as they often function as shibboleths for the variety under study, also with perception data, for instance by asking language users to describe their own language use in specific settings, and identifying features from their answers (cf. Lybaert, 2014b). It can then be investigated whether the use of these features correlates with external factors, like sociostylistic setting, speaker, gender, or age.

The above criteria (covariance, stylistic functions, idiovarietary elements, and emic category status) each offer an interesting perspective on the variety concept and form a catalog of features allowing empirical research into variety structure. In what follows, we present an empirical study conducted in Flanders, the northern Dutch-speaking part of Belgium, in which we use all of these criteria to evaluate their usefulness and the extent to which they yield converging or diverging outcomes. As present-day Flanders is known for its intriguing sociolinguistic dynamics, it constitutes an interesting laboratory to study the criteria under discussion.

## A flemish case-study

### Sociolinguistic background

The Flemish language repertoire is generally described as diaglossic (see for instance Grondelaers and Van Hout, 2011), to the extent that in between the standard language and the dialects, a whole continuum is found (usually subsumed under the header *tussentaal*, which literally means “in-between-language”)^3^. The standard is generally understood to be the Belgian Dutch standard, which corresponds in large measure to standard Dutch in the Netherlands (especially in its written form, cf. Grondelaers and Van Hout, 2011), but also deviates from it, especially phonetically (cf. Grondelaers et al., 2001; Vandekerckhove, 2005; Van De Velde et al., 2010). In its spoken form, the Belgian Dutch standard is also known as “VRT-Dutch” (Geeraerts, 1998) or “news broadcast Dutch” (cf. Plevoets, 2008), referring to the fact that the Flemish public broadcasting company, VRT, played a crucial role in the propagation of this standard. Even today, a rigorous norm is preserved (Vandenbussche, 2010), which, as far as formal registers are concerned, is strikingly more uniform than the spoken standard norm in the Netherlands (Grondelaers and Van Hout, 2011, p. 218). This VRT-Dutch is however often regarded as a virtual norm, expected or even imposed by authorities^4^, but rarely spoken in daily life (cf. De Caluwe, 2009, p. 19; Grondelaers and Van Hout, 2011).

**Figure 1:**
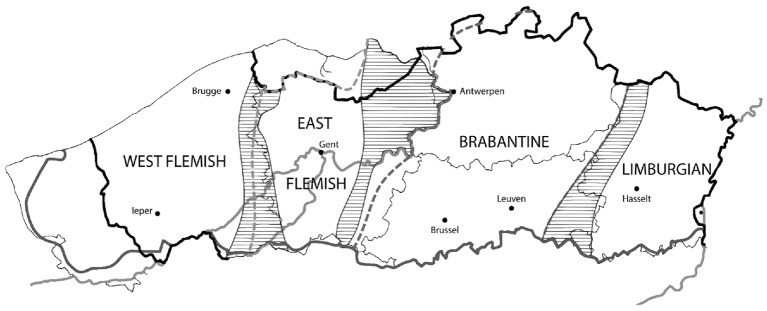
Dialect areas traditionally distinguished in Flanders (based on Taeldeman, 2009, p. 359).

On the opposite side of the spectrum, dialects are local varieties maximally distant from the standard. Traditionally, four major dialect areas are distinguished (cf. Figure 1), viz. West Flemish, East Flemish, Brabantic, and Limburgian (cf. Belemans and Keulen, 2004; Devos and Vandekerckhove, 2005; Ooms and Van Keymeulen, 2005; Taeldeman, 2005), which still appear to constitute relatively homogeneous areas from a sociolinguistic point of view, e.g., in terms of the amount of dialect leveling or the use of regional features in supraregional communication.

The language use in between dialect and standard, finally, is not only known as *tussentaal*, but also under denominators like *Soapvlaams* (Geeraerts, 1998, ‘soap-Flemish’), *Verkavelingsvlaams* (Van Istendael, 1989, ‘allotment Flemish’), or *Schoon Vlaams* (among others Goossens, 2000, ‘neat Flemish’). As pointed out above, *tussentaal* is a ‘mixed’ variety with elements from the standard language and local dialects and hardly any idiovarietary features, showing extensive regional variation. Yet, there are studies listing a number of ‘stable’ non-standard features that are either shared by most regional manifestations of *tussentaal* or expanding their use into regions in which they do not occur in the local dialects, and which allegedly constitute the heart of a homogenizing tendency (Rys and Taeldeman, 2007; Taeldeman, 2008; De Decker and Vandekerckhove, 2012). This homogenization, along with the observed functional elaboration of *tussentaal* at the expense of both standard language and dialect, is often interpreted as indicating changing (speech) norms in Flanders, which are analyzed in different ways (Ghyselen et al., 2016). Grondelaers et al. (2011) conclude from a speaker evaluation experiment that VRT Dutch is a virtual norm, and neither accented Dutch nor *tussentaal* function as prestige norms, which they interpret as a sign of *destandardization*. Jaspers and Van Hoof (2015, p. 35) argue that “the tension between standardizing and vernacularizing forces is intensifying and their relationship becoming more complex,” but interpret this as *late standardization* or *restandardization*, rather than destandardization, since VRT-Dutch clearly retains its social prestige in Flanders.

One of the crucial aspects of a scenario as sketched by Grondelaers et al. (2011) is that it assumes the emergence of a new variety which is taking over some of the functions of the traditional standard, VRT-Dutch. This article tries to answer the fundamental question whether a three-way distinction dialect-*tussentaal*-standard is grounded in an empirical reality. Even though this answer may depend on the context in which the investigation is carried out and may as such also show variation in Flanders, we use data from only one location, Ieper (but see Ghyselen, 2016b for a more comprehensive study). Ieper is located in the West-Flemish dialect area (cf. Figure 1)^5^, where the transition from a diglossic (dialects vs. standard) to a diaglossic repertoire (with intermediate usage) is believed to be in an early stage (Willemyns, 2007; De Caluwe, 2009; Ghyselen and De Vogelaer, 2013). This is a result of the fact that the linguistic repertoire in West Flanders is, in comparison to other regions, relatively rich, since the area is known to be fairly resistant to processes of dialect shift and dialect leveling (Willemyns, 2008; Ghyselen and Van Keymeulen, 2014), making it a particularly interesting methodological test case.

### Method

Since no corpora are available providing a comprehensive overview of situational variation in the speech of individual speakers of Belgian Dutch, a corpus was compiled between 2012 and 2014, comprising the speech of 10 highly educated women^6^ from Ieper (cf. Ghyselen, 2016a,b), of whom five were born between 1981 and 1986 and five between 1955 and 1961^7^. They were recorded in five speech settings: (1) a dialect test, (2) a standard language test, (3) a conversation with a friend^8^ from the same city, (4) a conversation with a friend from a different dialect area, and (5) a sociolinguistic interview with an unacquainted interviewer from a different dialect area. During the sociolinguistic interviews, data were gathered about the linguistic background of the informants and their perceptions of their own language use and language in Flanders in general. In the dialect and standard tests, the informants heard stimuli sentences spoken in either standard Dutch or in the local dialect, which they had to translate into the dialect of the elderly people in their town and standard Dutch “as heard during news broadcasts,” respectively. These tests were used to determine the informants' proficiency in the most acrolectal vs. basilectal speech styles available in a relevant location^9^. The recordings were transcribed orthographically using the Praat software (Boersma and Weenink, 2011)^10^; with the software package EXMARaLDA (Schmidt and Wörner, 2009) a searchable corpus of ~17 h of speech was built.

The corpus is analyzed using both quantitative and qualitative methods. Quantitatively, a correlative sociolinguistic approach is adopted: the distributions of 29 phonological and morphosyntactic features are studied in the five types of data, using both correspondence analyses and cluster analyses. These quantitative analyses are complemented with qualitative analyses of the interview data: the interview transcriptions were coded for 23 themes (e.g., informants' categorization of their own informal speech, definitions and evaluations of language varieties at play, attitudes toward specific variants, …).

In order to perform robust statistical analysis, frequent variables were selected, combining both very widespread and more regional features, since the geographical distribution of a dialect feature is known to have an important impact on its diachronic and stylistic dynamics (Schirmunski, 1928/1929; Taeldeman, 2009). With these criteria in mind, the following linguistic variables were studied (between brackets is their absolute frequency in the corpus):

- Realization verbal prefix <ge> in past participles (*n* = 1,076)
- Representation Standard Dutch [sχ] in anlaut (*n* = 27)
- Representation Standard Dutch [ε.i] (not before r or in auslautposition) (*n* = 2,161)
- Representation Standard Dutch [œ.y] (>wgm. û) (*n* = 937)
- Representation Standard Dutch [ɔ.u] before [t] of [d] (*n* = 255)
- Representation Standard Dutch [o:] (>ogm. au) before dental consonant (*n* = 222)
- Representation Standard Dutch [ɤ] (*n* = 5,642)
- Preservation of non-suffixal final schwa (*n* = 273)
- Representation Standard Dutch [o:] (>wgm û in open syllables) (*n* = 210)
- Representation of Standard Dutch initial /h/ in a selection of words (*n* = 1,720)
- t-deletion in *niet* (‘not’) or in *dat* (‘that’) + C (*n* = 3,870)
- t-deletion in *dat* (‘that’) + V (*n* = 983)
- Masculine singular indefinite article (*n* = 655)
- Verb form present simple 1st singular thematic verbs (in sentences without inversion; *n* = 793)
- Verb form present simple 1st singular athematic verbs (*n* = 366)
- Possessive pronoun 1 plural—form of pronoun (*n* = 222)
- Personal pronoun *he*—weak form in preverbal position (*n* = 264)
- Personal pronoun *he*—weak form in post-verbal position or after conjunctions (*n* = 314)
- Indefinite pronouns/adverbs (*n* = 359)
- Subject doubling 3 singular mascular/feminine, 1 plural, 3 plural in sentences with inversion and dependent clauses, with a strong pronominal subject (*n* = 284)
- Auxiliary in present perfect with *zijn* (‘to be’), *tegenkomen* (‘meet’), and *vallen* (‘fall’) as main verbs (*n* = 140)
- Subject doubling 2 singular/plural and 1 singular in sentences with inversion and dependent clauses, with a strong pronominal subject (*n* = 663)
- Preposition in subclauses with to-infinitives (*n* = 208)
- Expletive *dat* (‘that’) after conjunctions *wie, wat, waar, hoe, wanneer* en *of* (*n* = 359)
- Personal pronoun second singular, weak form in preverbal position (*n* = 489)
- Personal pronoun second singular, weak form in postverbal position (*n* = 502)
- Diminutives of nouns not ending in [t] (*n* = 244)
- Negative concord in sentences with *nooit* (‘never’), *niemand* (‘no one’), *nergens* (‘nowhere’) (*n* = 106)
- Possessive pronoun 1 plural—inflection before feminine singular nouns, masculine singular kinship terms, or plural nouns (*n* = 55)

In the Supplementary Material section, an overview can be found of the attested variants, along with information on their status in standard Dutch and the dialect of Ieper, and the codes used in the graphs of this paper. We refer to Ghyselen (2016b) for an in-depth discussion of the variants and the variable selection procedure.

To study how the attested variants correlate with each other and with the independent variables age, speech setting and speaker, a profile-based Correspondence Analysis (CA) was performed (cf. Plevoets, 2008, 2015; De Sutter et al., 2012). CA is a descriptive data analysis technique which studies correspondences or associations between rows and columns of a frequency table and “provides a detailed description of the data, yielding a simple, yet exhaustive analysis” (Costa et al., 2013, p. 1). The technique allows for the detection of potential clusters of linguistic features which behave alike, for instance clusters of dialect features or clusters of Standard Dutch features, and to visualize the structural distance (or the lack of a structural gap) between those clusters.

As a first step in correspondence analyses, two matrices with distances^11^ are calculated, one for the distances between columns (for instance the association between the situations ‘dialect test’ and ‘interview’ for the 66 attested language variants) and one for the distances between rows (for instance the association between the *ke*-diminutives and the *ge*-pronouns for the different situations and ages). A second step in the correspondence analysis is to plot the calculated distances in a two-dimensional space. For this purpose, the originally multidimensional matrices are reduced to two-dimensional ones using singular value decomposition, a dimension reduction technique which aims at preserving as much relevant information as possible. The distances from these two low-dimensional matrices are subsequently plotted in a biplot, in which the relative positions of the data points are indicative of their associations: variants plotted far away from each other are marked by low degrees of association; variants plotted close to each other show high associations. Important in the interpretation of correspondence plots are therefore the distances between data points and the way in which these cluster; the x- and y-axes do not have predetermined interpretations (cf. Geeraerts, 2010).

In this study, a profile based variant of CA was used. This profile based approach differs from ‘traditional’ CA in that the different language variants are not treated as autonomous data points, but as sublevels of a main variable. In our case, *ke*-diminutives and *je*-diminutives were, for instance, treated as sublevels of the variable ‘diminutive,’ and not as two autonomous variables. For more information on (the advantages of) this profile based approach, see Speelman et al. (2003) and De Sutter et al. (2012). Another aspect in which the correspondence analyses performed in this article differ from traditional CA, is that hypothesis-testing statistics were added; the technique was hence not purely descriptive. More specifically, confidence ellipses were drawn using bootstrap confidence interval construction (Reiczigel, 1996; Plevoets, 2013). These ellipses are interpreted in the same way as traditional confidence intervals (cf. Plevoets, 2013): only if ellipses of two categories (e.g., two age groups) do not overlap, the distance between those two categories is significant.

Correspondence analysis is closely related to cluster analysis, a descriptive multivariate technique which aims at identifying clusters in multivariate data in such a way that “the members of one group are very similar to each other and at the same time very dissimilar to members of other groups” (Gries, 2009, p. 337). While the strategy (grouping of similar categories by measuring co-variation) differs from correspondence analysis (projection onto a principal subspace), the results can be fairly similar: both methods are descriptive techniques which group variables based on their degree of correspondence (Lebart and Mirkin, 1993). In this paper, correspondence analysis is used as main analysis technique, because, unlike cluster analysis, it not only shows correlations between linguistic variables, but also with main effects such as age and situation. Since cluster analysis can be more convenient (Lebart and Mirkin, 1993, p. 15, remark that “it is much easier to describe a set of clusters than a continuous space”) and sometimes accounts for a much larger part of the original variance, the output of the correspondence analysis is used as input for cluster analyses, with the aim of facilitating the interpretation of the correspondence analyses. In these cluster analyses, the Ward-method, often also called the ‘minimum variance method,’ is used for clustering. This method, which has proven relevant in several linguistic studies (cf. Deumert, 1999; Gries, 2009), aims at minimizing the variance within each cluster (Janssens et al., 2008; Gries, 2009, p. 317). We moreover use bootstrap clustering (Suzuki and Shimodaira, 2006; Nerbonne et al., 2008), a variant of cluster analysis which first creates a large set of subsamples of the data (in our study 5,000) by combining random observations of the original dataset, and then generates a dendrogram for each of these datasets, resulting in a large number (again 5,000) dendrograms. These dendrograms are subsequently compared; clusters which occur in many versions (and hence have a high *bootstrap probability value*) can be considered more reliable than clusters which were only distinguished in a limited number of dendrograms. These probability values are of paramount importance in determining the number of relevant clusters, one of the biggest challenges in interpreting dendrograms (cf. Everitt, 1972).

## Results

### The overall repertoire

**Figure 2:**
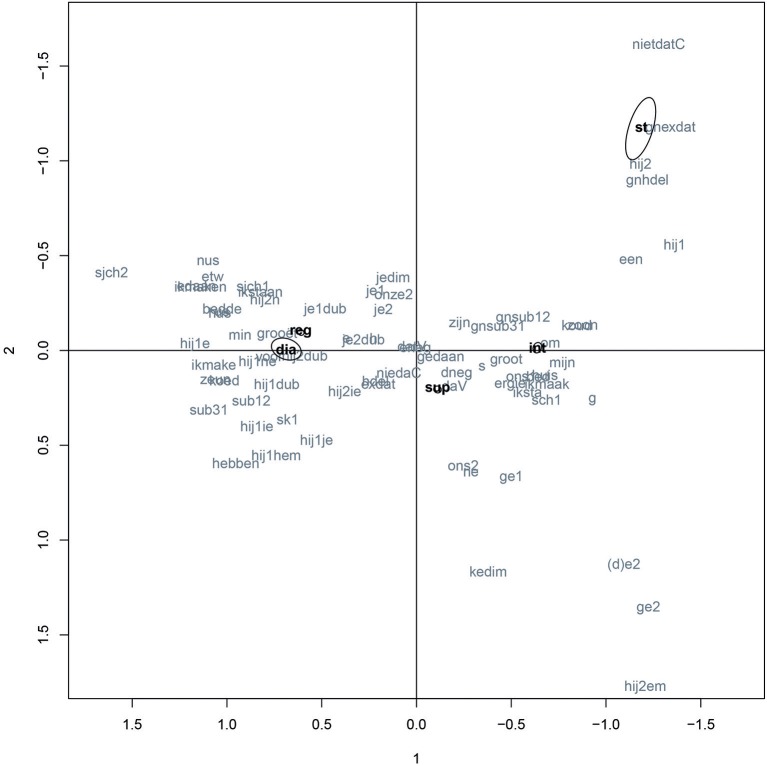
Correspondence plot Ieper with main effects for situation^14^.

Figure 2 shows the correspondence plot of all attested variants in Ieper (gray), with the main effects for situation (black)^12^. The two plotted dimensions in Figure 2 explain 54.3% of the original variance, which is fairly low; usually a total explained variance of 70–80% is aimed at (cf. Di Franco and Marradi, 2013, p. 83–84). While an analysis with four dimensions would be more suitable for our data from a statistical point of view (eigenvalues drop from the fifth dimension onwards; cf. Baayen, 2008, p. 130; Di Franco and Marradi, 2013, p. 83–84), it is difficult to visualize more than two dimensions comprehensibly^13^; the plots hence contain only two dimensions. Four dimensions will however serve as input for the cluster analysis.

The biplot translates different linguistic behavior into distances between data points. Thus, variants plotted close to each other, display similar behavior; large distances between variants imply weak correlations. In the top right corner of Figure 2, we for instance see a relatively small distance and hence a strong correlation between the realization of final -*t* in *niet* ‘not’ en *dat* ‘that (demonstrative)’ on the one hand (‘nietdatC’) and the absence of expletive *dat* ‘that (conjunction)’ on the other hand (‘gnexdat’). In contrast, a large distance can be seen between ‘nietdatC’ and ‘sjch2,’ the realization of sentence medial *sk* (in for instance *vissen* “to fish”) as [∫χ] or [∫], which indicates that these variants display very dissimilar behavior. A speaker realizing a sentence medial ^*^sk as [∫χ] or [∫] in a specific situation is in other words very unlikely to also realize final *t'*s in *niet* and *dat*.

As was mentioned in the Methods sections, the axes of the biplot do not have a predetermined interpretation. Meaning can, however, be uncovered by searching for patterns in the data points (Geeraerts, 2010, p. 244). In Figure 2, a clear horizontal continuum can be seen stretching from West-Flemish dialect variants on the left (e.g., the possessive pronoun *nus* ‘our’) to standard variants (e.g., ‘gnexdat’) and non-local dialect variants (such as the 2 sg. pronoun *ge*) on the right. The x-axis hence seems to be linked to locality (left: local, right: non-local). The y-axis is more difficult to interpret. In the top right corner, a cluster of features can be seen which are usually associated with formal standard language, such as the realization of final *t'*s (‘nietdatC’) and initial *h'*s (‘gnhdel’). In the bottom right corner, features are plotted which are not endogenous in the dialect of Ieper according to existing dialect descriptions (see e.g., FAND, 1998, 2000, 2005; MAND, 2005, 2009; SAND, 2005, 2007), but which are believed to be part of the homogeneising Flemish *tussentaal* (Taeldeman, 2008), such as *ke*-diminutives (‘kedim’) and the 2 sg. pronoun *ge* (‘ge1’ and ‘ge2’). The y-axis hence seems to be related to the type of non-dialectal language speakers target in non-local settings: from exogenously colored *tussentaal* in the bottom of the plot to VRT-Dutch on top, with in the center features occurring in both or in neither. Yet such an interpretation does not explain the distances between certain variants seen in the left of the biplot (e.g., between the possessive pronoun *nus* ‘our’ and the auxiliary *hebben* in the present perfect of the verbs *zijn, tegenkomen* en *vallen*), so there probably are other factors involved in this dimension, such as the differences between individual speakers.

When we look at the associations between the language variants on the one hand and the independent variable ‘situation’ on the other, a strong association (small distance) can be seen between the dialect test (‘dia’), the regional conversations between friends (‘reg’), and the dialect variants in the left of the biplot. The language use in the regional informal conversations hence differs only slightly from that in the dialect test and is fairly dialectal. The standard language test (‘st’) displays—as expected—strong associations with the standard language variants in the upper right corner of Figure 2. The large distance between the standard language test and the interviews (‘int’) shows that our speakers do not fully exploit their standard language competence during the interviews. This is consistent with the fact that Flemish speakers are known to feel uncomfortable about the VRT-Dutch norm (cf. Geeraerts, 2001 on the “sunday suit mentality”). In supraregional conversations with friends rather than an interviewer (‘sup’ in the graph) a much stronger association with the dialectal variants in the left of the graph is observed. Interestingly, this type of conversation also shows the strongest association with the non-standard, non-endogenous features in the bottom right of the graph, such as *ke*-diminutives, *ge*-pronomina, *ne*-articles and the unstressed form *hem* “him” as subject in inversion or subclauses.

On the basis of the results in Figure 2, we can conclude that the language repertoire of the Ieper informants constitutes a nice example of what Auer (2005) has labeled a diaglossic language repertoire, i.e., a repertoire marked by dialect, standard language and a continuum of intermediate forms. This overall diaglossic pattern should not, however, be taken as an indication that all individual speakers have diaglossic repertoires at their disposal; the overall continuous pattern might result from overlapping individual diglossic repertoires. It is beyond the scope of this paper to discuss all individual repertoires separately, but analyses reported in Ghyselen (2016a) indicate idiolectal variation in repertoire structure: whereas some speakers seem to have diaglossic repertoires (e.g., Wvla1, Wvla2, Wvla4, Wvlb1, Wvlb4), consciously realizing intermediate language use in supraregional informal settings, others have a more diglossic repertoire, switching between dialect and some form of (sub)standard Dutch (Wvla5, Wvlb2, Wvlb3, Wvla5, Wvlb5). Research with more speech settings and speech partners might reveal more clusters. In general, however, variation between repertoire types in Ieper hints at an ongoing transition from diglossia to diaglossia (Ghyselen, 2016a).

### Linguistic cohesion

In the following paragraphs, we examine whether varieties can be distinguished in Ieper's overall diaglossic repertoire by scrutinizing the variety criteria introduced earlier.

**Figure 3:**
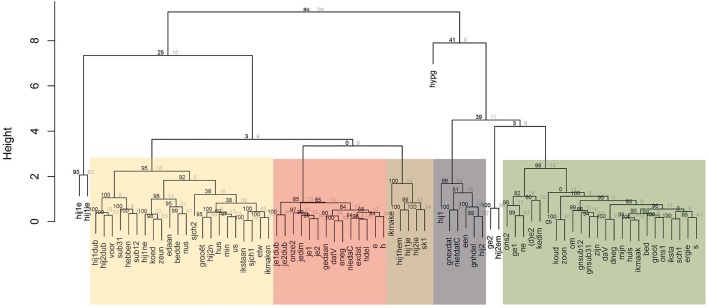
Dendrogram with variants attested in Ieper; cluster analysis on the basis of the four-dimensional coordinates for the different variants in a correspondence regression (bootstrap resampling: *n* = 5,000)^16^.

Can bundles of features be distinguished which strongly correlate in their socio-situative behavior? In the correspondence plot in Figure 2, several clusters of features can indeed be distinguished. To analyze these clustering tendencies more deeply, the first four dimensions of the correspondence analysis were used as input for a cluster analysis. Figure 3 shows the resulting dendrogram, which can be interpreted in terms of three clusters (with a cut-off point of 5), 7 (with a cut-off point of 3), or 10 (with a cut-off point of 2). AU *p*-values^15^ reported for every cluster can serve as guideline for the interpretation. Since cluster analysis is in essence a descriptive technique, the interpretation of a dendrogram also depends on theoretical concerns, however. For instance, clusters consisting of too few features are in our opinion not very relevant for an attempt to distinguish ‘varieties.’ With these considerations in in mind, five groups of features can be distinguished in Figure 3:

(1) A cluster (marked in yellow) with only dialectal features (such as *min* ‘my,’ the realization of wgm. î as [i] and non-suffixal schwa in words like *bed(de)* ‘bed’);
(2) A cluster (marked in red) with primarily dialect features, such as the indefinite article *e* (‘a’) and *h*-deletion (‘hdel’), but also some standard Dutch features, such as the realization of the initial consonant in past participles (*gedaan* ‘done’), and some features which are endogenous in both the Ieper dialect and the standard language, such as *je*-diminutives (‘jedim’) and *je* as 2sg. pronoun (‘je1’ and ‘je2’);
(3) A cluster (marked in brown) with principally eastern West-Flemish non-standard, non-endogenous dialect features, such as the suffix -*e* in the first person singular of thematic verbs in the present (‘ikmake’) or the realization of wgm. ^*^sk as [sk] in the anlaut (‘sk1’);
(4) A cluster (marked in gray) with only standard Dutch features, such as the absence of expletive *dat* (‘gnexdat’) or *h*-deletion (‘gnhdel’) and the realization of final *t*'s in *niet* and *dat* (‘nietdatC’).
(5) A cluster (marked in green) with primarily standard Dutch features (such as ‘bed,’ i.e., the lack of non-suffixal schwa), but also some non-standard, non-endogenous features, such as the *ge*-pronoun (‘ge1’) or *ne* as indefinite article (‘ne’).

**Figure 4:**
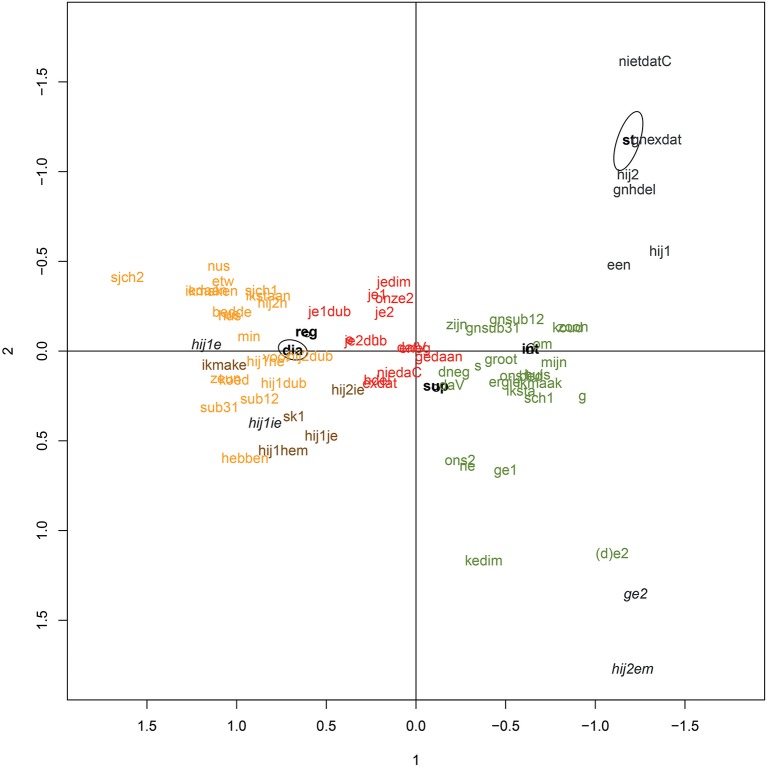
Correspondence plot Ieper with main effects for situation; the color codes indicate how the variants are categorized in a cluster analysis based on four-dimensional correspondence coordinates.

These five clusters are to a large degree supported by the data (AU *p*-value ≥ 85). The same goes for two other clusters in the dendrogram, namely the cluster with ‘hij1e’ and ‘hij1ie’ (AU value of 93) and the cluster with ‘ge2’ and ‘hij2em’ (AU value of 100), but given the low number of variants included in these clusters, these will be left aside. Interestingly, the distinguished clusters map nicely onto the two-dimensional correspondence plot (cf. Figure 4), indicating that the plot does offer an informative data-overview, despite the data reduction to only two dimensions.

It is important to highlight that the features within one cluster show strong correlations, but that they can also be combined with features from other clusters (be it with a different probability). As was already stressed above, correlation or covariance does not imply strict co-occurrence (Weinreich et al., 1968, p. 169). Moreover, it has to be kept in mind that, except for the standard language cluster (marked in gray), the clusters in the biplot in Figure 4 all show smooth transitions into one another.

### Stylistic functions

To investigate potentially routinized stylistic functions associated with variants in each of the distinguished clusters, we study when speakers use which cluster and complement these production data with qualitative interview data, as these yield more insight into the motives underlying the speech behavior.

The yellow cluster displays strong associations with both the dialect test and the regional conversations with friends for every speaker (Ghyselen, 2016a). If we assume that the cluster corresponds to what the informants name “the local dialect” in the interview, this cluster is the standard code for communication with other locals; that is after all how the dialect is described in the interviews. This type of language is moreover associated with coziness and familiarity (cf. Extract 1), which indicates that the dialect cluster functions as a regional informality marker.

(1) Interview Wvla5
  dialect? enkel hier thuis. in de streek. […] uh ma (maar) dialect is iets… ja. da (dat) ik spreek me (met) mensen da (dat) ik ken. die… die… uhm. ja. beetje. Ja uit dezelfde streek komen. uhm iets bekends. zo voelt et aan voor mij. *dialect? only here at home. in the area. […]. uhm but dialect is something… yes… that I speak with people I know… who… who… yes… kind of… yes come from the same area. uhm something familiar. that's how it feels to me*.

The brown cluster also shows strong associations with both the dialect test and the regional informal conversations but contains eastern West Flemish non-standard, non-endogenous variants^17^. The difference with the variants in the yellow cluster is not only that these features are not found in traditional descriptions of the Ieper dialect, but also that they are infrequent and not used by all speakers (cf. Table 1: mainly speakers Wvla1, Wvla3, and to a lesser degree also Wvlb1 use these features in their dialect). It seems likely that these features compare stylistically to the traditional Ieper dialect.

**Table 1:** Absolute frequencies of the eastern West-Flemish non-standard, non-endogenous variants (brown cluster).

**Variant**	**Number of attestations**	**Frequency variable**	**Situations**	**Speakers**
‘ikmake’	*N* = 34	*N* = 793	Dia (*n* = 9), reg (*n* = 19), sup (*n* = 6)	Wvla2 (*n* = 2), Wvla3 (*n* = 17), Wvla4 (*n* = 3), Wvla5 (*n* = 3), Wvlb1 (*n* = 4), Wvlb4 (*n* = 4), Wvlb5 (*n* = 1)
‘hij1hem’	*N* = 7	*N* = 264	Dia (*n* = 2), reg (*n* = 4), sup (*n* = 1)	Wvla1 (*n* = 1), Wvla3 (*n* = 4), Wvlb3 (*n* = 2)
‘hij1je’	*N* = 73	*N* = 264	Dia (*n* = 7), reg (*n* = 41), sup (*n* = 24), int (*n* = 1)	Wvla1 (*n* = 19), Wvla2 (*n* = 17), Wvla3 (*n* = 5), Wvla4 (*n* = 1) Wvlb1 (*n* = 8), Wvlb3 (*n* = 1), Wvlb4 (*n* = 1), Wvlb5 (*n* = 1)
‘sk1’	*N* = 1	*N* = 277	Reg (*n* = 1)	Wvla3 (*n* = 1)

The red cluster in Figure 4 seems to match what Wvla1 and Wvlb2 name *gekuist dialect* “cleaned-up dialect” when describing their language use in the supraregional informal conversations. The cluster does not occur in the personal repertoire of all speakers (cf. Ghyselen, 2016a), but for those speakers who use it, a strong association is indeed observed with the supraregional informal conversations, indicating that ‘cleaned-up dialect’ mainly functions as an informal, supraregional lingua franca (cf. Extract 2). From the interviews, it appears as if speakers of cleaned-up dialect have no intention to use the standard in supraregional informal conversations, and merely adapt their language for reasons of comprehensibility. This points toward a diaglossic language repertoire in the mind of the named speakers, who consciously realize something in between standard language and dialect.

(2) Interview Wvla1
  goh. ja vo (voor) de verstaanbaarheid uh spreek ik me (met) mensen die nie (niet) van West-Vlaanderen zijn wel ja. zo wa (wat) gekuister. (…) zeker geen standaardtaal […] ma (maar) 'k (ik) denk ook nie (niet) dat dad (dat) al tussentaal is. ma't (het) moe al wree (heel) officieel zijn voor da (dat) 'k (ik) echt…[Algemeen Nederlands probeer te spreken, ASG & GDV].
  *hmm. yes for reasons of comprehensibility uhm I speak somewhat more cleaned-up with people who are not from West-Flanders. (…) certainly not standard language or… […] but I don't think it is already tussentaal. […] but it has to be really official before I really… [try to speak standard Dutch, ASG* & *GDV]*.

The green cluster, which shows strong associations with the interview setting, seems to match ‘substandard’ language, as speakers in descriptions of their own language use labels like “attempted Standard Dutch” (Wvla1, Wvla2), “tussentaal” (Wvla2, Wvla3), “more like Standard Dutch” (Wvlb1), “the best Dutch I can realize” (Wvlb3), “standard language with an accent” or “something in the direction of Standard Dutch” (Wvla4), even though labels like “standard language” (Wvlb2) or, on the other hand, “not really standard, but a cleaned-up dialect version” (Wvlb5) are also found (cf. Extract 3). This language use has a double function: there is on the one hand a group of speakers who indicate using this intended standard Dutch as a lingua franca in all non-regional situations, whereas other speakers only rely on this type of language when a certain degree of formality is involved. There is also interpersonal variation in the degree to which non-standard, non-endogenous features are integrated in substandard language use (Ghyselen, 2016a). Those non-standard, non-endogenous features constitute a separate subcluster within the green main cluster (cf. “ons2,” “ge1,” “ne,” “(d)e2,” and “kedim” in Figure 3).

(3) Interview Wvla2
       Int    welk soort taalgebruik spreek je in dit interview?
  Wvla2    je beseft wel dat't (het) niet volledig AN is maar ge (je) probeert wel. […] je gebruikt da (dat) nie (niet) bewust… ma (maar) 't (het) is 't (het) feit da (dat) je 't (het) nie (niet) vlot de standaardtaal spreekt… […] waardoor da (dat) je de tussentaal gebruikt.
       Int    which kind of language do you speak in this interview?
  *Wvla2    you realize it's not completely Standard Dutch, but you do try. […] you do not speak this way deliberately… but it's mainly the fact that you do fluently speak standard language…[…] which causes you to speak tussentaal*.

The gray cluster in Figures 3, 4 shows strong associations with the standard language test for all speakers (cf. Ghyselen, 2016a). This cluster consists of standard features only, which are primarily realized in the standard language test, and to a much lesser degree in the interview setting. On the basis of quotes such as the ones in Extract 4 and the strong association with the non-spontaneous standard test, we could label these variants as characterizing a mainly virtual standard language norm, which is associated with the media and rarely realized in everyday life. We can however not exclude that there are other settings, not studied here, in which the speakers do exploit their standard language competence to the full. Both speaker Wvla4 and speaker Wvla1 for instance name presentations as one of the few speech settings in which they try to speak “real standard language” (Wvla4). The gray cluster hence potentially functions as a professionalism marker.

(4) Interview Wvla3
  Der zijn mensen die perfect Algemeen Nederlands kunnen ma (maar) da's (dat is) nie (niet) de normale […] iedereen probeert dan Algemeen Nederlands te spreken ma vo (voor) mij is da (dat) altijd een tussentaal en… […] ok misschien Martine Tanghe die op't journaal presenteert dat die Algemeen Nederlands spreekt daar kan ik mee z…. ma (maar) da (dat) vin (vind) ik nie (niet) dat da (dat) gesproken wordt in… in… in België op straat.
  *there are people who have mastery of perfect Standard Dutch but that's not the normal… […] everyone tries to speak Standard Dutch then…but to me that's always a tussentaal and… and… […] ok maybe Martine Tanghe who presents the news broadcast that she speaks Standard Dutch that is some I ag… but that is in my opinion not what is spoken in… in… in… Belgium in the street*.

In sum, we can say that the five clusters are not in a one-to-one relation with the five situations under study. The gray cluster—which we can dub VRT-Dutch—displays clear associations with the standard language test, but in the case of the other clusters, there is a functional overlap. The dialect (yellow cluster) and what we could call ‘horizontally leveled dialect’ (brown cluster) for instance both show strong associations with both the dialect test and the regional informal conversations, marking regional informality. Whether these clusters also function more generally as regionality markers, and hence are also used in formal regional settings, is a question for further research. In supraregional informal conversations some speakers realize cleaned-up dialect (red cluster), whereas others switch to a form of substandard (green cluster), which is used by all speakers in the interviews and functions as formal supraregional language; for some speakers as supraregional language *tout court*. Informants report comprehensibility as the main reason to use both cleaned-up dialect and substandard.

### Idiovarietary elements

A third matter to be discussed here is whether the distinguished clusters are marked by idiovarietary elements, i.e., language features which occur in one cluster only. While these are not essential for variety status (cf. supra and Berruto, 2010, p. 236), they do make a variety more recognizable. In the present study, it was investigated quantitatively whether specific language variants occur frequently in one type of setting only. In addition, metadata were checked for features which the informants named as typical of a certain variety, despite the fact that this issue was not brought up explicitly in the interviews.

The gray cluster, VRT-Dutch, is marked by several idiovarietary elements. That cluster displays strong associations with the standard language test for all speakers, and contains several variants (almost) exclusively bound to that situation, such as initial [h], *-t* in *niet* ‘not’ *not* and *dat* (‘that’), and the lack of expletive *dat* ‘that’ (≥80% of the potential cases)^18^. The realization of final consonants, which includes –*t* in *niet* “not” and *dat* “that,” is also consciously associated with standard language in the interviews (e.g., by Wvlb3 and Wvla5). Speaker Wvla3 reports that standard language should be spoken as it is written, which implies that final consonants should also be pronounced, as should initial [h].

Both the Ieper dialect (yellow cluster) and the horizontally leveled dialect (brown cluster) seem to contain several idiovarietary elements. Typical for the dialect of Ieper is for instance the suffix -*en* in the 1sg. singular –*en* following thematic verbs (‘ikmaken’) and word initial [∫χ] (‘sjch1’); horizontally leveled dialect is marked by the 1 sg. suffix -*e* (‘ikmake’). These variants disappear in the supraregional conversations and interviews. In the metadata, no statements are found concerning any of the named variants. The speakers indicate more generally that they consider dialectal vocabulary and to a lesser degree also accent as typical of the dialect, but they seldom give specific examples. Morpho-syntactic features were never mentioned (cf. also Lybaert, 2014b, p. 197).

The variants in the cleaned-up dialect cluster occur in various speech settings and hence do not seem to be of an idiovarietary nature. Variants such as expletive *dat* and *t*-deletion are also heard in the regional informal conversations and the interviews. When speakers mention the cleaned-up dialect in the interviews, no idiovarietary elements are mentioned either. They only indicate ‘cleaning up’ their dialect a little bit.

The green cluster, the substandard, does seem to be marked by idiovarietary features, such as the *ke*-diminutive, the *ne*-article, the uninflected 1pl. possessive form *ons* ‘our’ for feminine, masculine or plural nouns, and ‘*m* ‘him’ as a subject pronoun in the third person singular following conjugations or verbs. These features set apart the substandard from both dialect, VRT-Dutch and cleaned-up dialect. These idiovarietary features, however, do not occur in the substandard of all speakers, and are not mentioned in the interviews. Their occurrence does provide evidence for the existence of a distinct supraregional, informal variety.

A difficult question to tackle is why certain features end up being ‘idiovarietary’ and others do not. This question is closely related to the salience problem—why are speakers more aware of certain features than of others?—and the question why certain features are more prone to stylistic and diachronic variation than others. These questions have however not been convincingly answered up till now (cf. Kerswill and Williams, 2002; Ghyselen, 2016b, p. 305–347); many factors (of linguistic, social, and cognitive nature) have been observed to interact, and it proves difficult (not to say impossible) to predict which factor prevails in a specific setting.

### Emic category status

Finally, we also address the question whether the dataset offers evidence for emic category status of the observed clusters. Do the participants perceive the observed clusters as separate systems? To answer that question, we study the metadata in the sociolinguistic interviews. In the sociolinguistic interviews, all speakers mentioned dialect and standard as two extremes of the Flemish language repertoire. These extremes are perceived to be separate systems, which is for instance clear from Extract 5. No speaker distinguishes however between ‘real Ieper dialect’ (yellow cluster) and horizontally leveled dialect (brown cluster).

(5) Interview Wvlb4
  West-Vlaams is eigenlijk een aparte taal. […] mijn moedertaal. en dat uh Nederlands een aangeleerde taal is.
  *West-Flemish is actually a separate language. […] my mother tongue. and uhm Dutch a taught language*.

Concerning the clusters between dialect and standard Dutch, there are only two speakers who sketch a purely diglossic dialect-standard language image. All other speakers testify to the idea that in between the ‘real dialect’ and the ‘real standard’ other types of language are to be found (cf. Extracts 2, 3, and 4 above). Three speakers for instance mention a “nicely cleaned-up form of dialect.” Remarkably, these are three of the four speakers who also show strong associations with that cluster in their own production. Except for the two speakers with a diglossic perception, all speakers seem to be conscious of the substandard, which—as we already remarked above—gets various labels: “attempted Standard Dutch” (Wvla1, Wvla2), “tussentaal” (Wvla2, Wvla3), “more like Standard Dutch” (Wvlb1), “the best Dutch I can realize” (Wvlb3), “standard language with an accent,” or “something in the direction of Standard Dutch” (Wvla4). Thus, both cleaned-up dialect and substandard seem to have emic category status, although the perceptions are less uniform than those of dialect and standard language. This can be explained in linguistic terms—the intermediate clusters are less clearly defined and subject to more idiolectal variation—but also in social terms, with informants lacking the necessary metalanguage to describe the intermediate variations.

### Summary

The analyses above (cf. Table 2) provide several arguments to view dialect and VRT-Dutch as separate varieties in the language repertoire in Ieper: we can distinguish two separate clusters of variants marked by linguistic cohesion, clear stylistic functions, idiovarietary features, and emic category status. Within the dialect variety, two speech layers can be distinguished: the traditional dialect and a form of horizontally leveled dialect. These cannot be considered separate varieties as they do not have separate emic category status and their stylistic functions seem identical.

**Table 2:** Summary variety criteria.

	**Dialect**		**Intermediate usage**		**VRT-Dutch**
	**Local dialect of Ieper**	**“Horizontally leveled dialect”**	**Cleaned-up dialect**	**Substandard**	**VRT-Dutch**
Linguistic cohesion	+	+	+	+	++
Stylistic functions	Regional informality marker (Overlap with 2)	Regional informality marker (Overlap with 1)	Supraregional informality marker (Overlap with 4)	Suprareregionality marker (Overlap with 3)	Virtual norm? Professionalism marker
Idiovarietary elements	+	+	−	± (the subcluster with idiovarietary non-standard, non-endogenous features are not used by all speakers)	+
Emic category status	± (“dialect”)	± (“dialect”)	± (speaker dependent)	+	+

In addition to the dialect and VRT-Dutch, the substandard can be considered a separate variety as well: a bundle of features was observed which showed strong associations with relatively formal speech settings such as a sociolinguistic interview and for some speakers also with supraregional informal speech. Interestingly, the cluster is marked by a number of non-standard, non-endogenous features, which function as idiovarietary elements. It has to be stressed, however, that not all speakers realize these features to the same extent. It hence seems logical to distinguish two speech layers or formal types within the substandard: a type with non-standard, non-endogenous features, and a type without. Those speech layers do not constitute separate varieties—as was also the case for ‘traditional dialect’ and ‘horizontally leveled dialect’—given that the clusters are perceived as one category by the speakers and also strongly overlap functionally.

One can debate the status of the ‘cleaned-up dialect’ cluster, which on the one hand displays a fairly high degree of linguistic cohesion and has clear stylistic functions (comprehensible communication in supraregional informal settings), but, on the other hand, is only used (and recognized) by a limited number of speakers in this study. Rather than engaging in a moot debate on the status of ‘cleaned-up dialect,’ it has to be acknowledged that this outcome is a logical consequence of our implementation of several criteria for variety status, most of which are gradable rather than binary (e.g., linguistic cohesion, stylistic functions). This not only holds on the population level (e.g., some varieties may only be distinguished by a subgroup of speakers), but also on the level of the individual speaker (bundles of features can be considered clear or more doubtful instances of varieties). Cleaned-up dialect therefore represents a less prototypical instance of a variety, and illustrates our theoretical point that the variety concept is not a black-and-white notion.

More importantly than the debate on the status of ‘cleaned-up dialect,’ the data undeniably show that the traditional tripartite distinction dialect-*tussentaal*-Standard Dutch is difficult to substantiate empirically for the location under study. A pure continuum model is equally unsatisfactory, since it does not take into account the four ‘focal points’ that can be distinguished in the variation space emerging from our data. If one relaxes the notion ‘variety’ to include non-prototypical instances like cleaned-up dialect, a four-way divide, which distinguishes between two types of *tussentaal*, seems to match Ieper's linguistic reality best: it is both sociolinguistically and psycholinguistically accurate. Interestingly, at some points our criteria converged as to how the variation landscape is structured, in that many speakers having a variety at their disposal that was not observed across the board (e.g., ‘cleaned-up dialect’), were also the ones who mentioned it during the sociolinguistic interviews. Whether this is coincidental or not, is an issue for further research, as is the question whether similar results would be achieved if more ‘degrees of freedom’ (e.g., in the social profile of informants, test settings, …) would be included in the study.

## Discussion and conclusion

At the beginning of this paper, we raised the question whether systems or varieties can be distinguished in the heterogeneity of everyday language. This question was shown to be relevant for many concepts in contemporary linguistics, such as code-switching vs. style-shifting or multilingualism vs. translanguaging, which build on implicit or explicit assumptions about the structure of underlying linguistic variation. Taking stock of criteria traditionally used for variety status—such as homogeneity, stylistic functions, emic category status, and idiovarietary features—we argued that these form a catalog of criteria which can be tested against empirical data. We especially emphasized the importance of factoring in the ontological status of production patterns, as this criterion allows distinguishing categories which are not only statistically, but also cognitively real. Ensuing from the proposed multidimensional perspective on varieties is a flexibilization of the variety concept: in line with a cognitive view on categorization, whether a type of language can be considered a variety is a matter of degree, depending on the number of variety characteristics displayed by that language use. The variety question does not have a hard and fast, universal answer; insight into variety structure can only be achieved through close empirical scrutiny of production and perception patterns in both individual language users and a given language community. Combining quantitative and qualitative approaches, the case-study presented here focused on stylistic variation in Dutch as spoken in Ieper, in the Belgian province of West Flanders, by a relatively homogeneous and small group of test persons. The data seem to show that the West Flemish speech repertoire, while diaglossic in nature, showed four “focal points,” which could be labeled varieties. These include a fairly stable dialect variety, a more or less virtual standard Dutch variety, and two intermediate varieties which we labeled ‘cleaned-up dialect’ and ‘substandard.’

Some tentative diachronic conclusions can be drawn from the data, too. In all likelihood, much of the variation in our data can be understood as indicative of ongoing change. At the beginning of this paper, processes of dialect leveling, dialect shift, destandardization, and demotization were discussed, and shown to yield proper predictions on the level of both production and perception. It is beyond the scope of this paper to discuss the age effects in the production data elaborately, but one striking result is that a clear overall age effect could only be found for the interview setting, showing stronger associations with the VRT-Dutch cluster for the older than for the younger speakers in this setting. This age effect implies standard language change, but is ambiguous as to its interpretation as an instance of destandardization or demotization. The perception data indicate that a scenario of destandardization is not very likely, however, given that all informants reproduced many aspects associated with a Standard Language Ideology in the interviews, i.e., the idea that one type of language is inherently better than other types of language. All informants also indicated aiming at Standard Dutch in many settings. Thus, it seems likely that the variants produced more frequently by younger informants during the interview, such as *t*-deletion, *ne*-articles and expletive *dat*, are increasingly accepted within the Standard Dutch norm. Interestingly, no age effects were found in the dialect test setting, nor in the informal regional conversations, indicating that the Ieper region is fairly resistant to processes of dialect leveling and dialect shift (at least with respect to the studied variables).

On a theoretical level, the case of Ieper shows that even in situations in which a linguistic repertoire presents itself as a sociolinguistic continuum, it remains worthwhile to try identifying focal points, thus acknowledging that linguistic variants are organized in structures. Our conclusion does not imply that the very concept of diaglossia is superfluous, however, if only because clear differences can be seen with diglossic repertoires. The Ieper case also provides no principled argument against the possibility that other linguistic repertoires can indeed display a more fluid, continuum-like structure. The empirical approach adopted here has the advantage that it avoids projecting preconceived structure or even uniformity on the sociolinguistic landscape, and links linguistic variants to social categories in a bottom-up way. This makes the methods suitable to tap into the social meaning of variants and even map out how social categories are structured *in*, but also *by* language. This is an important asset in an era in which the “third wave” in sociolinguistics (Eckert, 2012) is aiming at more precision in determining the social concerns expressed in language, and increasingly conceiving of language variation as producing social differentiation rather than simply reflecting it. The methods also take into account behavior of individual language users. Johnstone (2000) discusses such interest in the “individual voice” in language against the background of a larger shift toward a more phenomenological approach to language and greater particularity in methods for its study. Rather than zooming in on the particular, the methods adopted here allow to study the behavior of linguistic individuals while still enabling us to derive a generalization on the level of the speech community. As such, this shows that approaches conceiving of language from the perspective of the individual vis-à-vis the social may be less fundamentally different than suggested in Johnstone's account. Indeed, a current convergence is observed between social and cognitive sciences, which manifests itself in the rise of new fields such as social neuroscience, and, within linguistics, in new frameworks in both psycholinguistics and sociolinguistics, such as sociolinguistic cognition and cognitive sociolinguistics (see De Vogelaer et al., 2017, p. 19–21 for discussion).

Finally, on a methodological level, analyses like the ones in this article require data incorporating not only more social and regional variation, but also stylistic variation along other parameters than regionality and formality. While the methods for such a ‘big data’ analysis of language variation are being rapidly developed, corpora including enough spoken data to analyze the full spectrum between dialects (or other colloquial varieties) and standard language, remain unavailable for many speech communities, such as Flanders. We hope that the presented research can form an impetus toward larger-scale investigations into variety structure.

## Ethics statement

This study was carried out in accordance with the recommendations of the ethical commission of the UGent Faculty of Arts and Humanities with written informed consent from all subjects. All subjects gave written informed consent in accordance with the Declaration of Helsinki.

## Author contributions

This article is based on an empirical investigation designed and carried out by A-SG under the supervision of GD. The theoretical framework adopted and the interpretations described in the paper were intensely discussed, and are shared by both authors. A-SG did the bulk of the writing and GD critically revised the manuscript.

### Conflict of interest statement

The authors declare that the research was conducted in the absence of any commercial or financial relationships that could be construed as a potential conflict of interest. The reviewer MD and handling Editor declared their shared affiliation.

## Footnotes

^1^A related issue is the distinction between code-switching and borrowing, which typically also draws on sharp boundaries between linguistic systems, which need not be cognitively real, however. See Matras (2009, p. 110–114) for discussion.

^2^Cf. Rys (2007), who observed that Flemish children acquiring dialect as a second language realize only 8% of the 13.824 studied tokens in a picture naming task hyperdialectally. Considering that hyperforms are to be expected in this test context (more than in spontaneous speech settings or among native speakers of dialect), this is a low number.

^3^In fact “tussentaal” was coined as a term to discredit the particular intermediate varieties as an imperfectly acquired standard variety (Taeldeman, 1992), which, in line with naming practice in second language acquisition research, would translate as “interlanguage.” The term seems to be increasingly adopted by laypeople unfamiliar with this rather negative connotation, who in many settings consider the variety an attractive alternative to both the standard variety, which is deemed overly serious or stiff, and the dialects, which are considered rude or old-fashioned (Lybaert, 2014a).

^4^See for instance Van Hoof and Vandekerckhove (2013) on language policy in Flemish media, and also Delarue (2016) on language policy in Flemish education.

^5^This area roughly corresponds to the province of West-Flanders, the westernmost province in Flanders, but the boundaries do not completely coincide.

^6^For reasons of feasibility, we decided to keep the sex and education level of the speakers constant. In line with the study's focus on recent developments relating to standardization, we opted for highly educated women, as (1) women have been reported to be the leaders of change (Labov, 1990) and (2) a higher education is typically associated with more mobility and intenser supraregional contacts (cf. Britain, 2011, p. 54 on “middle-class mobilities”).

^7^Speakers of the younger age group have the letter “a” in their speaker code (e.g., wvla1), whereas the older speakers have the letter “b” (e.g., wvlb1).

^8^Gender was not controlled for in these conversations, as it was already difficult finding suitable informants without constraints on the gender of the speech partners. Six of the 20 conversations with friends (of the same or of a different dialect area) were mixed sex conversations; the majority were same sex conversations.

^9^The data obtained in the test settings are of a very different nature than spontaneous speech data (cf. Lenz, 2003, p. 57–62). This difference will be taken into account when analyzing the results.

^10^Of each conversation with a friend 30 min were transcribed; the interviews and dialect and standard tests were transcribed entirely.

^11^Given that we are dealing with frequency tables, the distances are calculated using chi-square metrics.

^12^Supplementary Table 2 lists the absolute and relative frequencies of the non-standard variants per situation.

^13^The package “corregp” (Plevoets, 2015) does allow three-dimensional plotting, but the 3D-plots for our data turned out to be poorly interpretable because of the large number of plotted variants.

^14^For theoretical reasons, the axes are mirrored (with on the first dimension positive values for instance left and negative values right). As such, the visual representation of the data matches traditional representations of dialect-standard continua better.

^15^Distances between datapoints were calculated using Euclidean distance measures; distances between clusters with Ward's method.

^16^The R-package pvclust (Suzuki and Shimodaira, 2013) generates a bootstrap probability value (BP) and an approximately unbiased probability value (AU) for each cluster. The BP-value is obtained by traditional bootstrapping, in which no demands are made on the size of the subsamples, whereas AU-values are calculated by means of *multiscale bootstrapping*, in which the size of the subsamples is systematically altered. Given that AU-values are more reliable according to Suzuki and Shimodaira (2006), we will use these values as guideline when interpreting the output of the dendrogram. AU-values of 95% or larger—corresponding to a *p*-value of 0.05 or smaller—indicate highly reliable clusters.

^17^One can debate the status of the masculine preverbal pronoun *je* in the third person singular in the Ieper dialect. Bille (2009, p. 128) does not name the form in her description of the Ieper dialect, but on the basis of present empirical dialect research, it is difficult to delineate clear areas for the dialectal pronouns *je, ‘n, ne, enne* and *e*.

^18^The variants did occur in the interviews too, but were low frequent in this setting (maximally in 38% of the possible cases). That the variants also occur in the interview setting is in our view not a reason to not consider these forms as typical of VRT-Dutch; exclusivity is after all too severe a criterion when defining idiovarietary features. Furthermore, the language use in the interview setting is for many speakers an attempt toward VRT-Dutch. Some speakers approach this target more closely than others, which explains why we can find some attestations of idiovarietary features of VRT-Dutch in the interview setting.
