# Supplementary material for: Seeking Systematicity in Variation: Theoretical and Methodological Considerations on the “Variety” Concept
Source: Front Psychol. 2018 Mar 26;9:385. doi: 10.3389/fpsyg.2018.00385 (PMC5879321; doi:10.3389/fpsyg.2018.00385)
Supplement: Supplementary file 1 [file DataSheet1.pdf]

Supplementary Material

Seeking Systematicity in Variation: Theoretical and Methodological Considerations on the ‘Variety’ Concept

Anne-Sophie Ghyselen*, Gunther De Vogelaer

*** Correspondence:** Corresponding Author: annesophie.ghyselen@ugent.be

Supplementary Table 1. Overview studied variables and variants (*=variant is typical of traditional dialect in Ieper, **=typical of Standard Dutch, ***= does not occur in traditional dialect nor in the standard, ****=occurs in both the traditional dialect and the standard)

Variable	Attested variants (‘code’[1]) + type[2]	Example
PHONETIC/PHONOLOGICAL VARIABLES		
**Realization verbal prefix <ge>** in past participles (n=1076)	> deletion first consonant (‘edaan’) *<br>> realization first consonant (‘gedaan’) **	[æ]daan, [ə]daan (‘done’)<br>[ɣə]daan
**Representation Standard Dutch [sχ]** in anlaut (n=277)	> [ʃχ] (‘sjch’) *<br>> [sχ] (‘sch’) **	[ʃχo:lə] (‘school’)<br>[sχo:l]
**Representation Standard Dutch [ɛ.i]** (not before r or in auslautposition) (n=2161)	> short monophthong (‘min’) *<br>> long monophthong or diphthong[3] (‘mijn’) **	[mɪn] ('mine')<br>[mɛ:n], m[ɛ.i]n
**Representation Standard Dutch [œ.y]** (> wgm. û) (n=937)	> short monophthong (‘hus’) *<br>> long monophthong or diphthong (‘huis’) **	[hys] ('house')<br>[hœ.s]<br>[hœ.ys]
**Representation Standard Dutch [ɔ.u]** before [t] of [d] (n=255)	> short monophthong (‘koed’) *<br>> long monophthong or diphthong (‘koud’) **	[stut] ('naughty')<br>[stɔ.t]<br>[stɔ.ut]
**Representation Standard Dutch [o:]** (> ogm. au) before dental consonant (n=222)	> diphthong (‘grooët’) *[4]<br>> long monophthong (‘groot’) ****	[ɣruət] ('big')<br>[ɣro:t]

---

1 These codes will be used in the graphs of this contribution.

2 This categorisation is based on data from SAND (Barbiers et al. 2005; Barbiers et al. 2008), FAND (Goossens et al. 1998; Goossens et al. 2000; De Wulf et al. 2005) and MAND (De Schutter et al. 2005; Goeman et al. 2008). For a number of variables, specialized dialectological descriptions were consulted (De Vogelaer 2008: on subject marking; Cornips & De Vogelaer 2009: on gender in Dutch; De Vogelaer & Vandenberghe 2006: on indefinite pronouns and adverbs). This a-priori classification of language variants does not necessarily correspond to laymen’s classifications of the variants; it merely offers an insight into how the variants are characterized in traditional dialect and standard language descriptions.

3 For this variable and also for the realization of Standard Dutch [œ.y] and [ɔ.u], no distinction was made between respectively the long monophthongs [ɛ:] , [œ.], [ɔ.] on the one hand and the dipthong [ɛ.i], [œ.y] and [ɔ.u] on the other, nor between closed and open variants of the diphthongs. Without acoustic analyses, those distinctions proved too difficult to make objectively.

4 In some areas of the research area, the diphthong is a typical feature of the dialect; in the city center of Ieper, however, the basilectal form is the long monophthong which also characterizes the standard language.

**Representation Standard Dutch [ɣ]** (n=5642)	> laryngalization (‘h’) *<br>> [ɣ] (‘g’) **	[h]oed [h]edaan ('well done')<br>[ɣ]oed [ɣ]edaan
**Preservation of non-suffixal final schwa** (n=273)	> with schwa (‘bedde’) *<br>> without schwa (‘bed’) **	*vrouwe* ('woman')<br>*vrouw*
**Representation Standard Dutch [o:]** (> wgm û in open syllables) (n=210)	> palatalized form (‘zeun’) *<br>> [o:] (‘zoon’) **	[zønə] ('son')<br>[zo:n]
**Representation of Standard Dutch initial 'h'** in a selection of words (n=1720)	> *h*-procope (‘hdel’) *<br>> realization *h* (‘gnhdel’) **	*oeveel* ('how much')<br>*hoeveel*
**t-deletion** in *niet* ('not') or in *dat* ('that') + C (n=3870)	> *t*-apocope (‘niedaC’) *<br>> realization final consonant (‘nietdatC’) **	*je moet da nie doen.* ('You do not have to do that')<br>*je moet dat niet doen.*
**t-deletion** in *dat* ('that') + V (n=983)	> *t*-apocope (‘daV’) ***<br>> realization final consonant[5] (‘datV’) ****	*da ook* ('that too')<br>*dat ook*
MORPHOSYNTACTIC VARIABLES		
**Masculin singular indefinite article** (n=655)	> *e* (‘e’) *<br>> *ne* (‘ne’) ***<br>> *een* (‘een’) **	*e vent* ('a guy')<br>*ne vent*<br>*een vent*
**Verb form present simple 1st singular** thematic verbs (in sentences without inversion) (n=793)	> infinitive[6] (‘ikmaken’) *<br>> root +*e* (‘ikmake’) ***<br>> root (‘ikmaak’) **	*ik spelen* ('I play')<br>*ik spele*<br>*ik speel*
**Verb form present simple 1st singular** athematic verbs (n=366)	> infinitive (‘ikstaan’) *<br>> root (‘iksta’) **	*ik zijn* (‘I am’)<br>*ik ben*
**Possessive pronoun 1 plural** – form of pronoun (n=222)	> *(n)us/(n)uze* (‘(n)us’) *<br>> *ons/onze* (code: ‘ons1’) **	*(n)us kind, (n)uze moeder* ('our child', 'our mother')<br>*ons kind, onze moeder*
**Personal pronoun 'he'** - weak form in preverbal position (n=264)	> *ne/e* (‘hij1(n)e’) *<br>> *je* (‘hij1je’) *<br>> *hij* (‘hij1’) **	*Ne komt ook*. ('he is coming too')<br>*Je komt ook*.<br>*Hij komt ook*. ('he is coming too')
**Personal pronoun 'he'** - weak form in postverbal position or after conjunctions (n=314)	> *‘n/ne* (‘hij2n’) *<br>> *em* (‘hij2em’) ***<br>> *ie* (‘hij2ie’) **<br>> *hij* (‘hij2’) **	*Komt ’n ook?* ('is he coming too?')<br>*Komt em ook?*<br>*Komtie ook?*<br>*Komt hij ook?*
**Indefinite pronoun/adverb** of person, matter or place (n=359)	> *etwien, etwat/etwuk, etwaarschen* (‘etw’) *<br>> *iemand, iets, ergens* (‘ergie’) **	*Is er etwat*? ('is something going on?')<br>*Is er iets?*
**Subject doubling** 3 singular mascular/feminine, 1 plural, 3 plural in sentences with inversion and dependent clauses, with a strong pronominal subject[7] (n=284)	> subject doubling (‘sub31’) *<br>> no subject doubling (‘gnsub31’) **	*A me wider komen*… (Lit: 'if we we come')<br>*Als wij komen*... (Lit: 'if we come')

[5] No distinction is made between the variants da[t] and da[d], since that distinction is often difficult to make without acoustic analyses.

[6] This infinitive form is widespread in Flanders in a few monosyllabic athematic verbs (a.o. *doen* 'do' en *gaan* 'go'), but the occurrence of the infinitive form in almost all verbs is confined to a small area in West-Flanders.

[7] In these cases, subject doubling with a weak pronoun is obligatory in the local dialect (De Vogelaer 2008: 326).

**Auxiliary** in present perfect with *zijn* ('to be'), *tegenkomen* ('meet') and *vallen* ('fall') as main verbs (n=140)	> *hebben* (‘hebben’) *<br>> *zijn* (‘zijn’) **	*Ik heb ziek geweest.* (Lit: 'I have ill been')<br>*Ik ben ziek geweest.* (Lit: 'I am ill been')
**Subject doubling** 2 singular/plural and 1 singular in sentences with inversion and dependent clauses, with a strong pronominal subject (n=663)	> subject doubling (‘sub12’) *<br>> no subject doubling (‘gnsub12’) **	*Morgen kom ek ik ook.* (Lit: 'tomorrow come I I too').<br>*Morgen kom ik ook.* (Lit: 'tomorrow come I too').
**Preposition** in subclauses with *to*-infinitives (n=208)	> preposition *voor* (‘voor’) *[8]<br>> preposition *om* (‘om’) **	*Dat kost veel voor te wassen.* (Lit: 'that costs much for wash')<br>*Dat kost veel om te wassen*. (Lit: 'that costs much to wash')
**Expletive *dat*** ('that') after the conjunctions *wie*, *wat*, *waar*, *hoe*, *wanneer* en *of* (n=359)	> with expletive *dat* (‘exdat’) *<br>> without expletive *dat* (‘gnexdat’) **	*Ik weet niet wie dat er komt* (Lit: 'I know not who that is coming')<br>*Ik weet niet wie er komt*. (Lit: 'I know not who is coming')
**Personal pronoun** second singular, weak form in preverbal position (n=489)	> *je komt* (‘je1’) ****<br>> *ge komt* (‘ge1’) ***<br>> *je komt gie/ge komt gie* (‘je1dub’) *	*je speelt* (‘you are playing’)<br>*ge speelt*<br>*je speelt gie*
**Personal pronoun** second singular, weak form in postverbal position (n=502)	> *kom je* (‘je2’) ****<br>> *kom je gie/kom de gij* (‘je2dub’) *<br>> *kom (d)e* (‘(d)e2’) ***<br>> *komt ge* (‘ge2’) ***	*speel je?* (‘are you playing?’)<br>*speel je gie?*<br>*speel de?*<br>*speelt ge?*
**Diminutives** of nouns not ending in [t] (n=244)	> *ke*-diminutive (‘kedim’) ***<br>> *je*-diminutive[9] (‘jedim’) *	*bloemke/bloemeke* ('little flower')<br>*bloempje*
**Negative concord** in sentences with *nooit* ('never'), *niemand* ('no one'), *nergens* ('nowhere') (n=106)	> double negation (‘dneg’) *<br>> single negation (‘eneg’) **	*Ik ga dat nooit nie doen*. (Lit: 'I go that never never do')<br>*Ik ga dat nooit doen*. (Lit: 'I go that never do')
**Possessive pronoun** 1 plural – inflection before feminine singular nouns, masculine singular nouns kinship terms, or plural nouns (n=55)	> no inflection (‘ons2’) ***<br>> with inflection (‘onze2’) ****	*ons moeder* ('our mother')<br>*onze moeder*

Supplementary Table 2. Absolute and relative frequencies non-standard variants per situation

Variabele	Dia[10]		Reg		Sup		Int		St	
	%	n[11]	%	n	%	n	%	n	%	n
**Realization verbal prefix <ge>** in past participles (n=1076)	28	56	23	359	2	362	0	240	0	59
**Representation Standard Dutch [sχ]** in anlaut (n=277)	100	2	92	110	23	86	0	79	0	0

[8] It can be debated whether the construction with *voor*-preposition is endogenous in the dialect of Ieper. See Ryckeboer (1983) for more information.

[9] The allomorphy within the *je*-suffix was not taken into account as this complicates the calculation of distance measures: some of the *je*-suffixes in the Ieper dialect coincide for instance with Standard Dutch *je*-suffixes (*bloemetje*, ‘little flower’), whereas others have another allomorph (*boeksje* versus *boekje*, ‘little book’).

[10] Shaded cells represent situations in which the variable was not attested.

[11] N: number of times the variable was attested.

**Representation Standard Dutch [ɛ.i]** (not before r or in auslautposition) (n=2161)	100	72	95	680	35	716	2	607	0	86
**Representation Standard Dutch [œ.y]** (> wgm. û) (n=937)	100	21	93	323	16	273	1	295	4	25
**Representation Standard Dutch [ɔ.u]** before [t] of [d] (n=255)	100	22	99	81	19	74	0	51	0	27
**Representation Standard Dutch [o:]** (> ogm. au) before dental consonant (n=222)	78	9	70	99	15	74	0	29	0	11
**Representation Standard Dutch [ɣ]** (n=5642)	82	129	96	2086	81	1960	58	1412	62	55
**Preservation of non-suffixal final schwa** (n=273)	86	21	94	89	3	89	0	64	0	10
**Representation Standard Dutch [o:]** (> wgm û in open syllables) (n=210)	100	20	97	65	21	57	0	48	0	20
**Representation of Standard Dutch initial 'h'** in a selection of words (n=1720)	97	87	98	705	88	560	63	291	14	77
**t-deletion** in *niet* ('not') or in *dat* ('that') + C (n=3870)	100	81	99	1259	99	1447	83	1010	8	73
**t-deletion** in *dat* ('that') + V (n=983)	0	3	5	328	15	342	11	308	0	2
**Mascular singular indefinite article** (n=655)	86	22	97	289	88	234	44	89	9	21
**Verb form present simple 1st singular** thematic verbs (in sentences without inversion) (n=793)	90	31	94	254	16	210	0	278	0	20
**Verb form present simple 1st singular** athematic verbs (n=366)			84	155	17	127	0	84		
**Possessive pronoun 1 plural** – form of pronoun (n=222)	100	8	98	49	23	89	0	67	0	9
**Personal pronoun 'he'** - weak form in preverbal position (n=264)	100	39	98	98	51	73	5	21	0	33
**Personal pronoun 'he'** - weak form in postverbal position or after conjunctions (n=314)	48	21	72	129	47	119	12	25	0	20
**Indefinite pronoun/adverb** of person, matter or place (n=359)			78	116	16	106	0	137		
**Subject doubling** 3 singular mascular/feminine, 1 plural, 3 plural in sentences with inversion and dependent clauses, with a strong pronominal subject (n=284)			74	73	29	86	1	95	0	30
**Auxiliary** in present perfect with *zijn* ('to be'), *tegenkomen* ('meet') and *vallen* ('fall') as main verbs (n=140)	4	25	51	35	22	36	0	15	0	29
**Subject doubling** 2 singular/plural and 1 singular in sentences with inversion and dependent clauses, with a strong pronominal subject (n=663)	100	3	93	131	71	160	6	359	0	10

**Preposition** in subclauses with *to*-infinitives (n=208)	80	5	89	70	40	77	4	47	0	9
**Expletive *dat*** ('that') after the conjunctions *wie, wat, waar, hoe, wanneer* en *of* (n=359)	100	12	99	113	98	131	62	93	20	10
**Personal pronoun** second singular, weak form in preverbal position (n=489)	0	11	17	206	49	198	56	64	0	10
**Personal pronoun** second singular, weak form in postverbal position (n=502)			12	228	32	192	34	82		
**Diminutives** of nouns not ending in [t] (n=244)	0	13	8	100	62	73	13	15	0	23
**Negative concord** in sentences with *nooit* ('never'), *niemand* ('no one'), *nergens* ('nowhere') (n=106)			3	34	2	52	5	20		
**Possessive pronoun** 1 plural – inflection before feminine singular nouns, masculine singular nouns kinship terms, or plural nouns (n=55)			12	8	43	30	23	17		

References

Barbiers, Sjef , Johan Van der Auwera, Hans Bennis, Eefje Boef, Gunther De Vogelaer & Margreet van der Ham. 2008. Syntactische atlas van de Nederlandse dialecten. Deel II. Amsterdam: Amsterdam University Press.

Barbiers, Sjef, Hans Bennis, Gunther De Vogelaer, Magdalena Devos & Margreet Van der Ham. 2005. Syntactische atlas van de Nederlandse dialecten. Deel I: Pronomina, congruentie en vooropplaatsing Amsterdam: Amsterdam University Press.

Cornips, Leonie & Gunther De Vogelaer. 2009. Variatie en verandering in het Nederlandse genus. Taal en Tongval 22.1-12.

De Schutter, Georges, Boudewijn Van den Berg, Ton Goeman & Thera De Jong. 2005. Morfologische Atlas van de Nederlandse Dialecten. Deel I Amsterdam: Amsterdam University Press.

De Vogelaer, Gunther. 2008. De Nederlandse en Friese subjectsmarkeerders: geografie, typologie en diachronie Gent: Koninklijke academie voor Nederlandse taal- en letterkunde.

De Vogelaer, Gunther & Roxane Vandenberghe. 2006. *Iemand* of *entwie*, *ergens* of *ieveranst*. Een taaltypologisch perspectief op onbepaalde voornaamwoorden en bijwoorden in de Zuid-Nederlandse dialecten. Structuren in talige variatie in Vlaanderen, ed. by J. De Caluwe & M. Devos, 91-113. Gent: Academia Press.

De Wulf, Chris, Jan Goossens & Johan Taeldeman. 2005. Fonologische Atlas van de Nederlandse Dialecten. Deel IV: De consonanten Gent: Koninklijke Academie voor Nederlandse Taal- en Letterkunde.

Goeman, Ton, Marc Van Oostendorp, Piet Van Reenen, Oele Koornwinder, Boudewijn van den Berg & Anke Van Reenen. 2008. Morfologische Atlas van de Nederlandse Dialecten. Deel II Amsterdam: Amsterdam University Press.

Goossens, Jan, J. Taeldeman, G. Verleyen & C. de Wulf. 1998. Fonologische Atlas van de Nederlandse Dialecten. Deel I: Het korte vocalisme. Gent: Koninklijke Academie voor Nederlandse Taal- en Letterkunde.

Goossens, Jan, Johan Taeldeman & Geert Verleyen. 2000. Fonologische atlas van de Nederlandse dialecten II - III. Deel II: De Westgermaanse korte vocalen in open syllaben. Deel III :De Westgermaanse lange vocalen en diftongen Gent: Koninklijke Academie voor Nederlandse Taal- en Letterkunde.

Ryckeboer, Hugo. 1983. *Voor te* + infinitief. Verkenning naar de dynamiek van een dialectisme. Taal & Tongval 35.83-89.
